# Anglo-Swiss Milk Food

**Published:** 1885-05

**Authors:** 


### Anglo-Swiss Milk Food.

The attention of the medical profession has of late been closely directed to the cause of the enormous proportion of infantile deaths, as recorded in the mortuary records. The general conclusion seems to be that this terrible loss of life in young children, especially at the critical period during the earlier months of dentition, (at which time some artificial food is generally either added to, or entirely substituted for the natural aliment), is largely due to innutrition; in other words, that owing to the want of an artificial food calculated to supply the great demands upon the system, the infant is, in effect, starved.

Although, perhaps, diluted cow's milk offers the best substitute for that designed by Nature, yet in so few cases can this be found pure, at least in cities, that it does not contain the essential ingredients for the support and development of infantile life.

Estimated in a cursory manner, human milk contains about 890 parts of water to 110 parts solid matter; and of this solid matter caseine, fat and saccharine matter occupy the larger proportion, it is assimilated by the infant, and we have as a result healthy growth and development; but if these constituents are wanting, the child is imperfectly nourished, and easily falls a victim to the many disturbances which accompany dentition. To meet this want several artificial Milk Foods, more or less scientifically prepared, have been introduced to the public, and one of the most desirable is that known as the Anglo-Swiss Milk Food (made by the Anglo Swiss Condensed Milk Company at Cham, Switzerland.) This Food has been proved to contain all the necessary ingredients for a reliable Food for infants, and having received the highest endorsements from the medical profession in Europe and in New York and other large American cities, may, therefore, be used with perfect confidence by all having the care of young children.—*Miss. Medical Monthly.*

